# Supplementary material for: Interrogating the degradation pathways of unstable mRNAs with XRN1-resistant sequences
Source: Nat Commun. 2016 Dec 5;7:13691. doi: 10.1038/ncomms13691 (PMC5150221; doi:10.1038/ncomms13691)
Supplement: Supplementary Information — Supplementary Figures 1-8 and Supplementary References [file ncomms13691-s1.pdf]

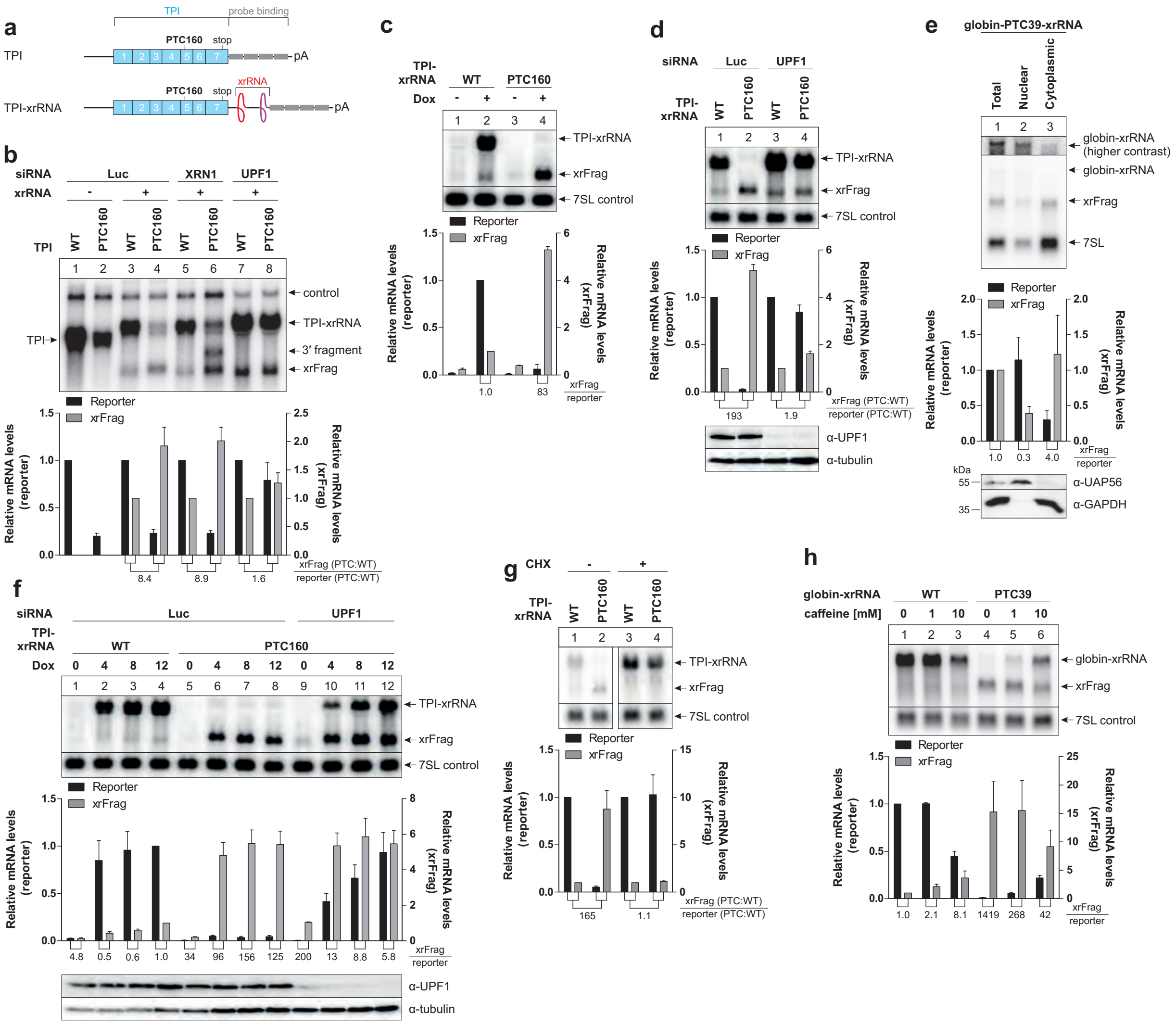

Supplementary Figure 1

NMD-dependent accumulation of xrFrags derived from reporter mRNA degradation

(a) WT or PTC-containing TPI reporter mRNAs with or without xrRNA are depicted as in Fig. 1. (b-h) Northern blots of total RNA extracted from transiently transfected HeLa cells (b) or stable cell lines (c-h) expressing the indicated reporter mRNAs. Co-transfected LacZ or endogenous 7SL served as control RNA. Mean values of reporter and xrFrag signal ± s.d. (n = 3) were quantified and for each knockdown or treatment condition the PTC values were normalized to the WT. For sub-cellular fractionation (e), the values for reporter and xrFrag mRNA levels were normalized to the respective value of the total RNA fraction. The ratio of xrFrag to reporter mRNA levels is indicated below all graphs. Successful fractionation was confirmed by the nuclear marker UAP56 and the cytoplasmic marker GAPDH (d, f) Western blot analysis of total protein extracted from the same samples with UPF1 antibody, tubulin served as loading control.

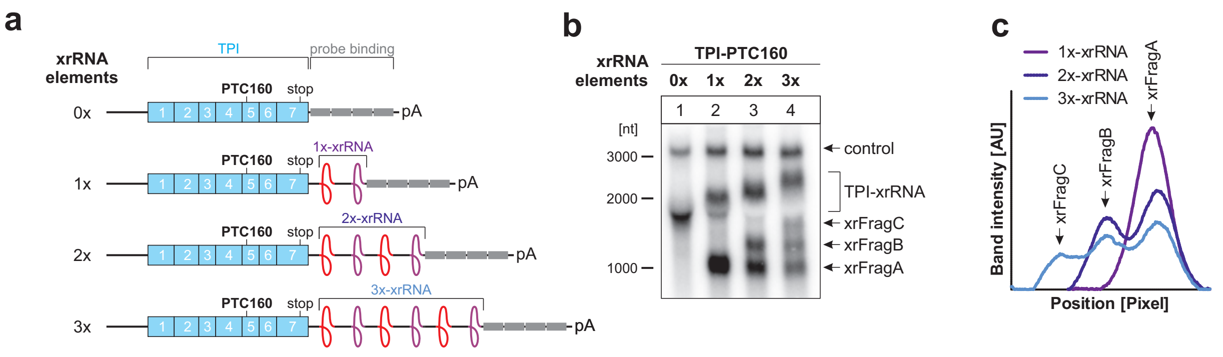

Supplementary Figure 2

Degradation of xrFrags via 5′-3′ decay

(a) Schematic representation of the PTC-containing TPI reporter mRNAs as in Fig. 1, indicating the multiple xrRNA element repeats. (b) Northern blot of total RNA extracted from transiently transfected HeLa cells expressing the indicated reporter mRNAs. Co-transfected LacZ served as control RNA. (c) Lane profile of the xrFrags shown in (b), indicating the position of xrFragA, B and C.

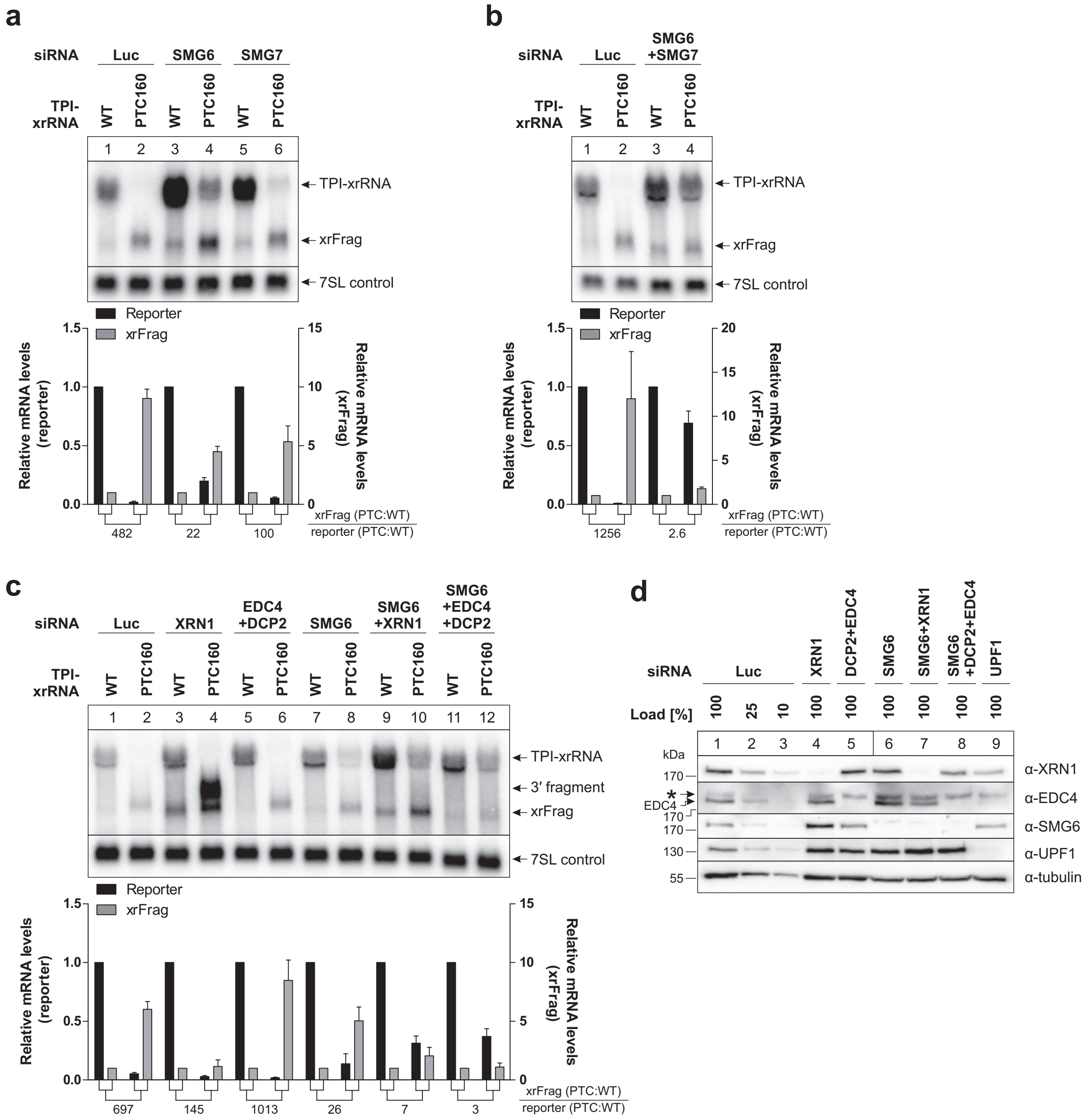

Supplementary Figure 3

NMD utilizes the whole spectrum of degradation pathways

(a-c) Northern blots of RNA samples extracted from stable HeLa cell lines transfected with the indicated siRNAs and expressing the indicated reporter constructs. Endogenous 7SL served as control RNA. Mean values of reporter and xrFrag signal ± s.d. (n = 3) were quantified and for each knockdown condition the PTC values were normalized to the WT. The ratio of xrFrag to reporter mRNA levels is indicated below the graph. (d) Western blot analysis of knockdown efficiencies was done with the indicated antibodies, tubulin served as loading control.

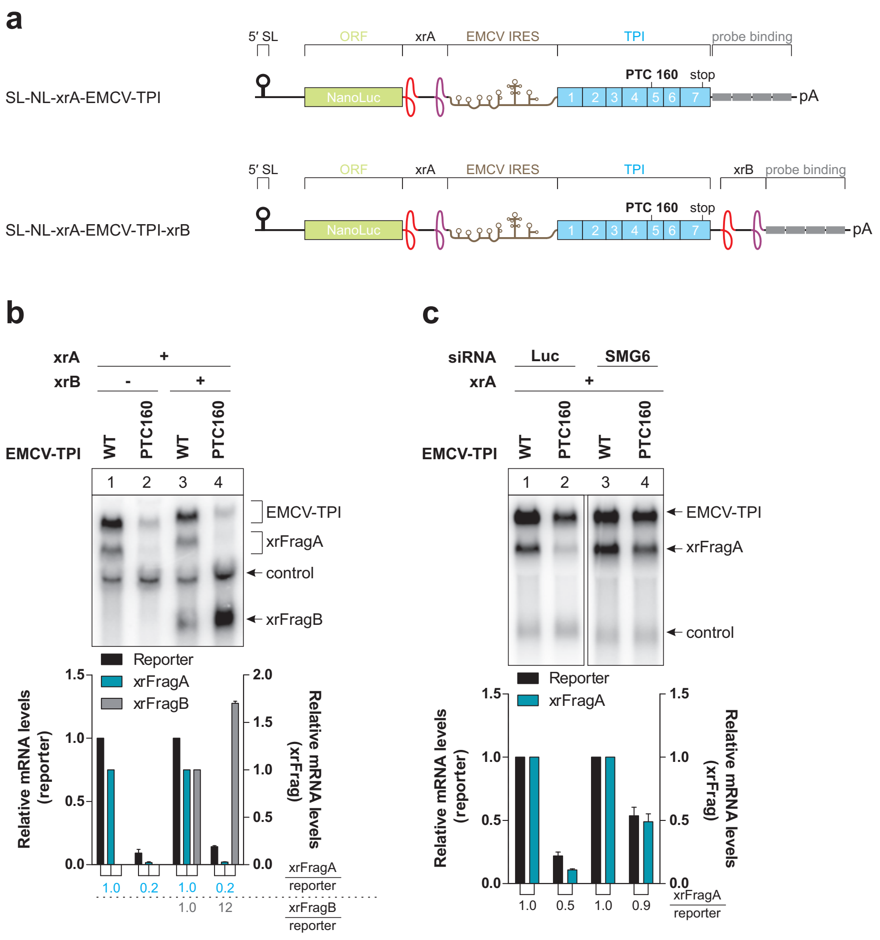

Supplementary Figure 4

Degradation of IRES-containing dual xrRNA reporter via SMG6-catalyzed endocleavage

(a) Schematic representation of the dual xrRNA IRES reporter as in Fig. 1. (b,c) Northern blots of RNA samples extracted from HeLa cells transfected with the indicated siRNAs and reporter constructs. Co-transfected TPI (b) or globin WT (c) served as control mRNA. Mean values of reporter and xrFrag signals ± s.d. (n = 3) were quantified. The ratio of xrFrags to reporter mRNA levels is indicated below the graph.

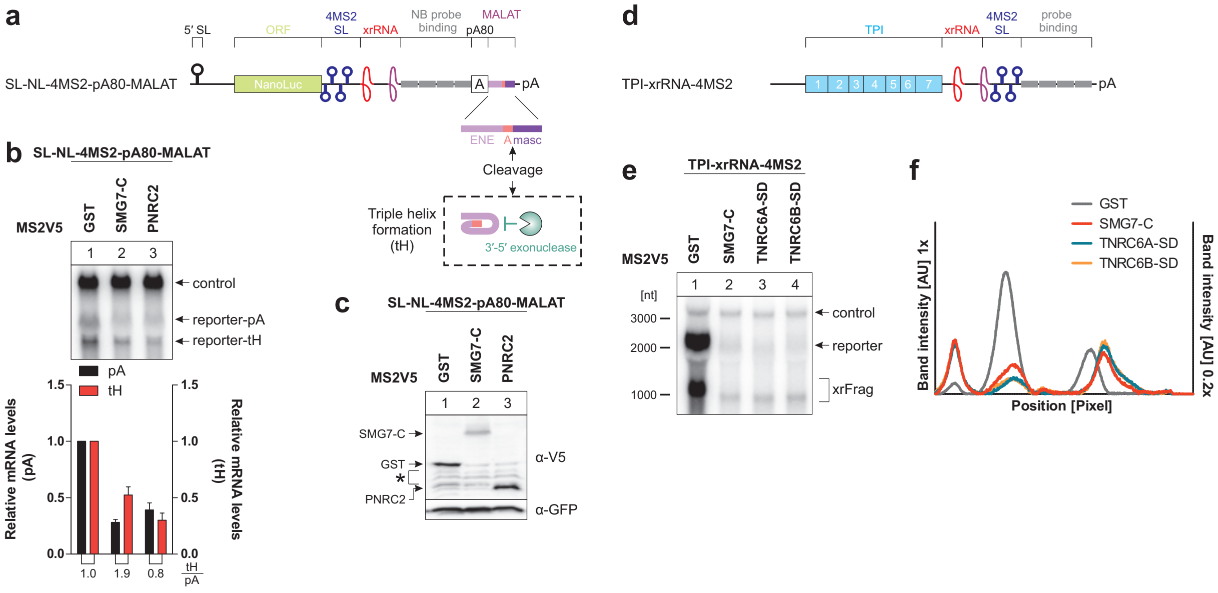

Supplementary Figure 5

Tethered SMG7 induced deadenylation-dependent degradation of reporter mRNAs

(a) Schematic representation of the non-translatable NanoLuc tethering reporter containing a 3′ MALAT sequence. To stimulate transport to the cytoplasm 80 template-encoded adenosines (pA80) were inserted upstream of the MALAT sequence. The processing by endonucleolytic cleavage of masc RNA and subsequent triple helix formation is indicated. (b) Northern blot of RNA samples extracted from HeLa cells transfected with the indicated tethering constructs and the SL-NL-4MS2-pA80-MALAT reporter. Mean values ± s.d. (n = 3) for polyadenylated reporter (pA) and triple helix reporter (tH) levels were quantified and normalized to tethered GST, which served as control. The ratio of tH:pA mRNA levels is indicated below the graph. (c) Western blot showing the expression levels of the MS2V5-tagged constructs with GFP serving as transfection control. Unspecific bands are indicated with asterisks. (d) Schematic representation of the TPI-xrRNA-4MS2 tethering reporter with inverted xrRNA and 4MS2 elements. (e) Northern blot of RNA samples extracted from HeLa cells transfected with the indicated tethering constructs and the TPI-xrRNA-4MS2 reporter, LacZ served as control. Approximate RNA size is indicated on the left. (f) Lane profiles of the northern blot in (e), with size-normalized LacZ control serving as reference point. Due to the differences in reporter and xrFrag intensities, the values for GST tethering were plotted on the left axis (1x intensity), for SMG7-C, TNRC6A-SD and TNRC6B-SD on the right axis (0.2x intensity).

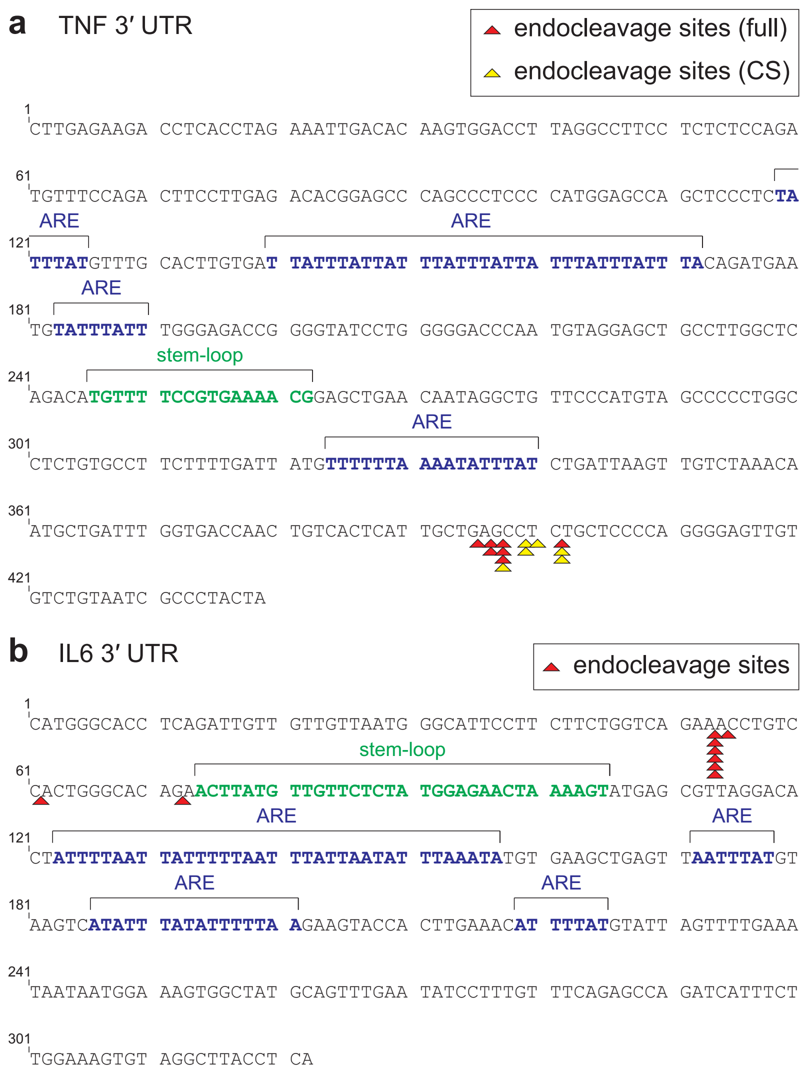

Supplementary Figure 6

Annotated sequences of cytokine elements

(a, b) The DNA sequence of the TNF-α and IL6 element used in reporter constructs are shown with annotations of sequence motifs. Single or overlapping AREs are annotated according to AREsite2[1]. Red and yellow triangles represent cleavage sites identified by sequencing of individual unique cloned 3′ fragments.

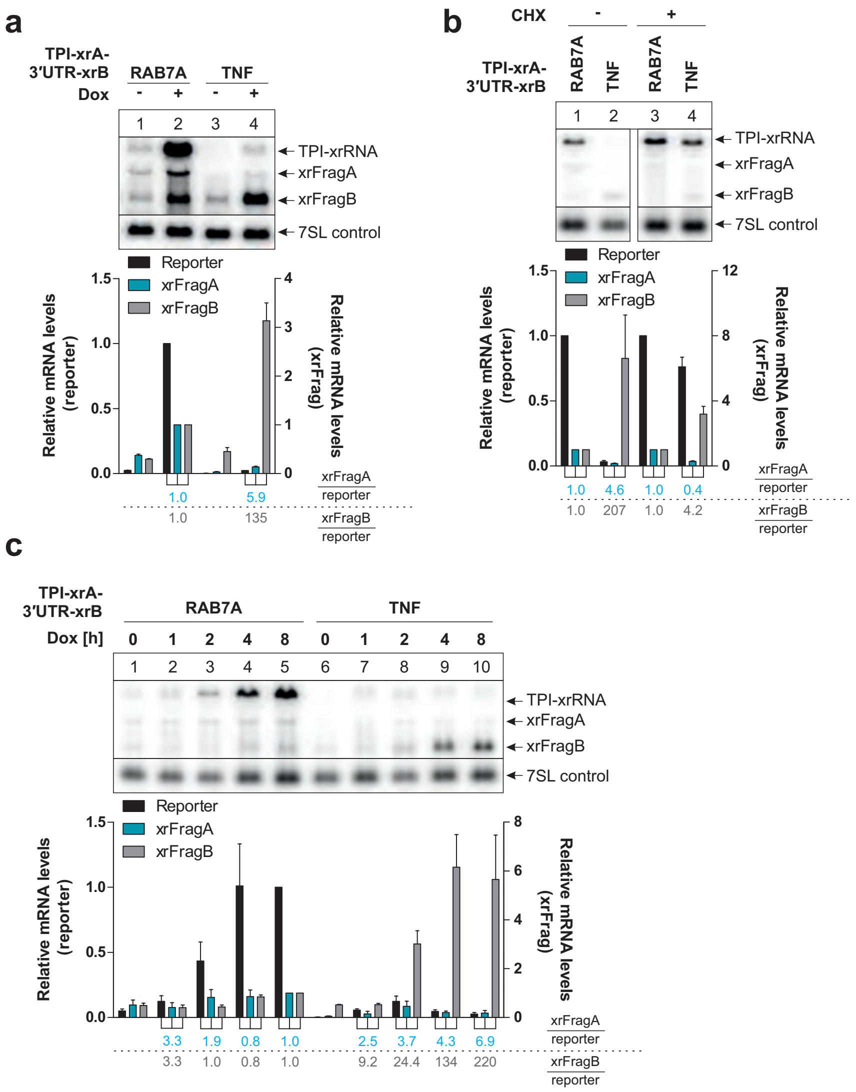

Supplementary Figure 7

Detection of endocleavage within the TNF-α 3′ UTR framed by two xrRNA elements

(a-c) Northern blots of total RNA extracted from stable HeLa cell lines expressing the indicated dual xrRNA reporter mRNAs. Mean values of reporter and xrFrag signal ± s.d. (n = 3) were quantified and for each knockdown condition the PTC values were normalized to the WT. The ratio of xrFrag to reporter mRNA levels is indicated below the graph.

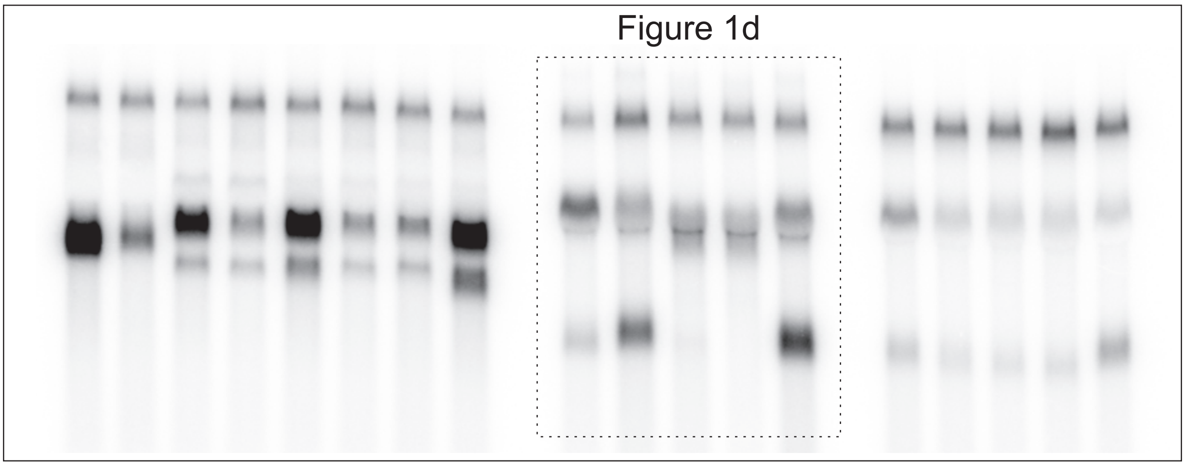

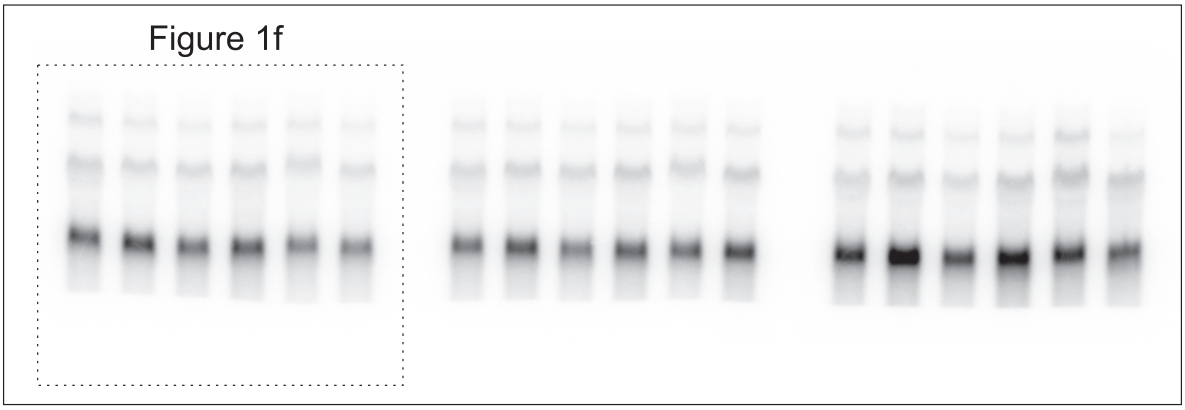

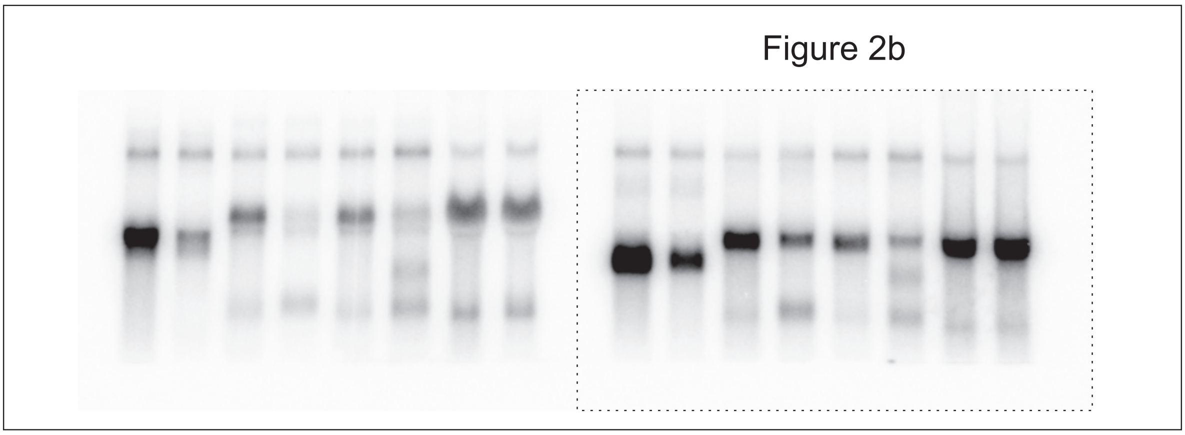

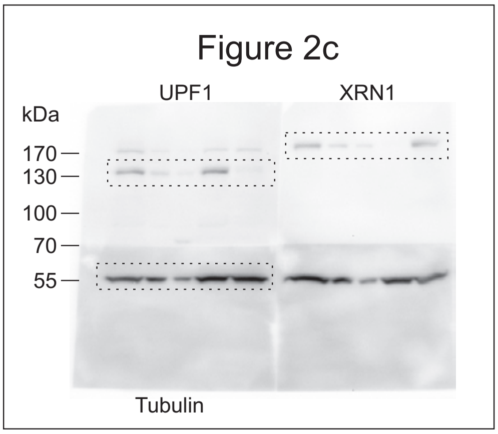

Supplementary Figure 8

Uncropped blots of Figures 1-8 as indicated.

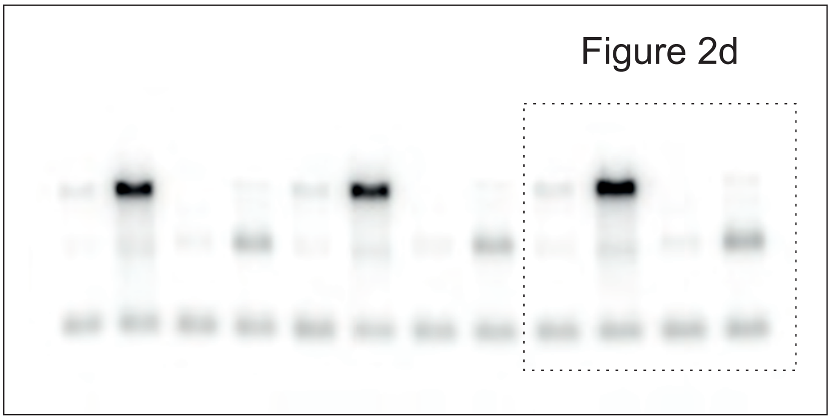

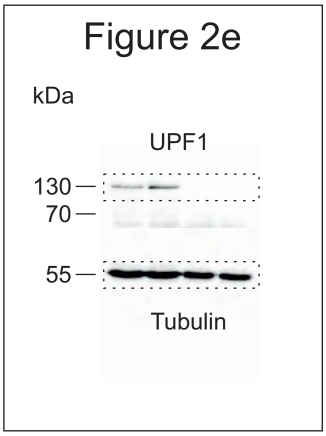

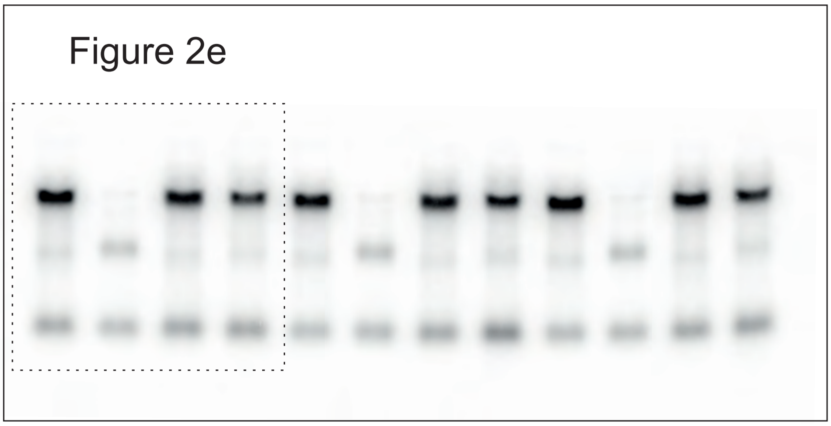

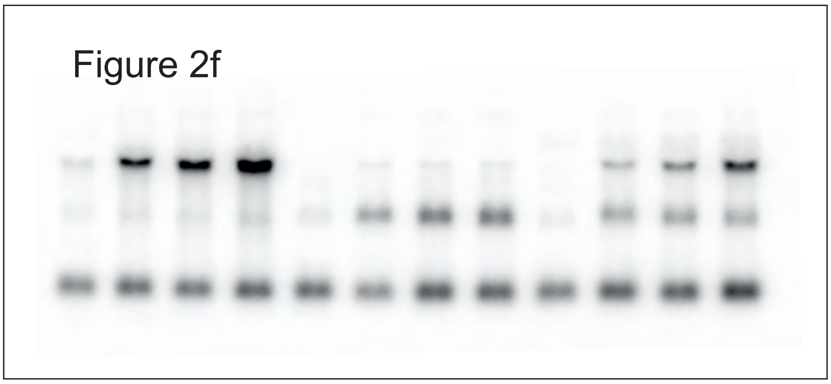

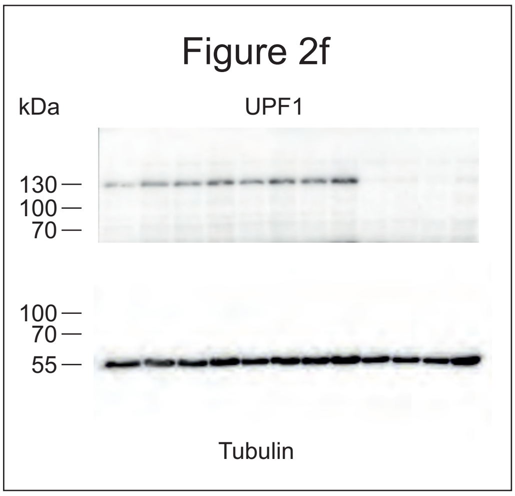

Supplementary Figure 8, continued

Uncropped blots of Figures 1-8 as indicated.

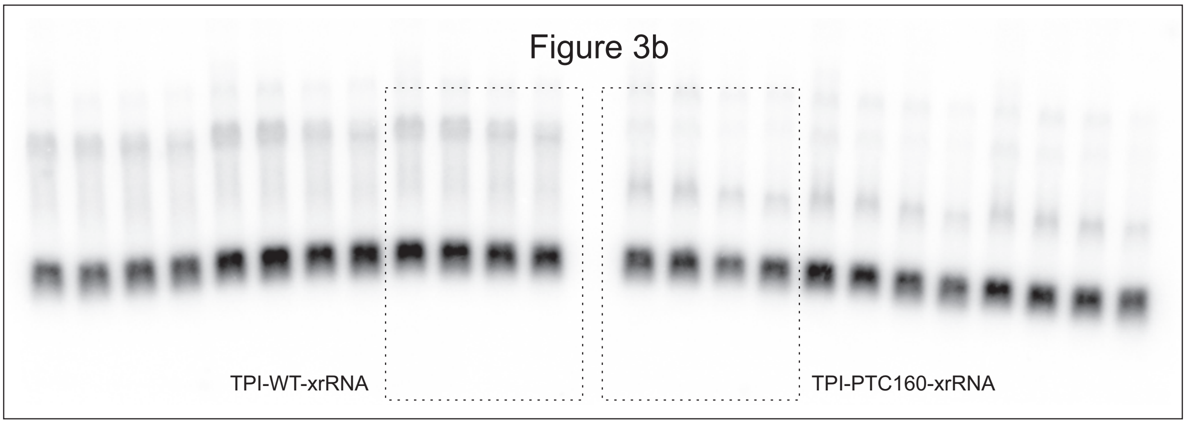

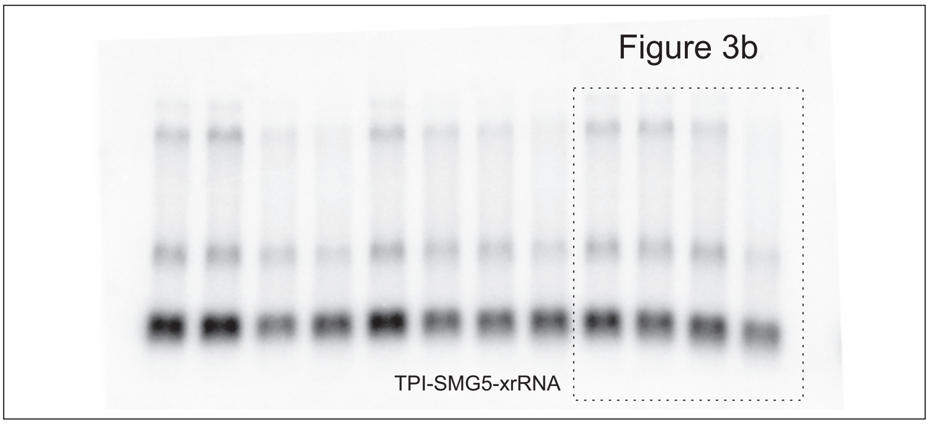

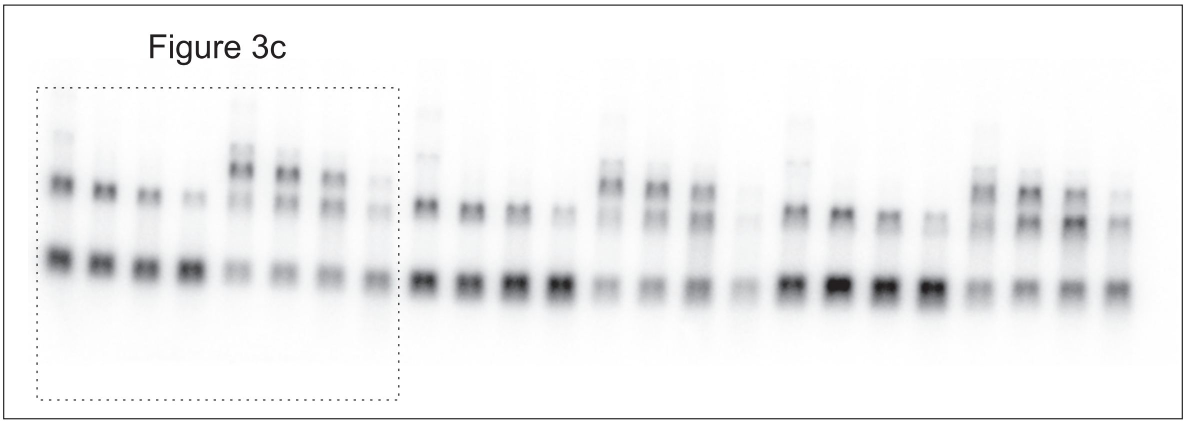

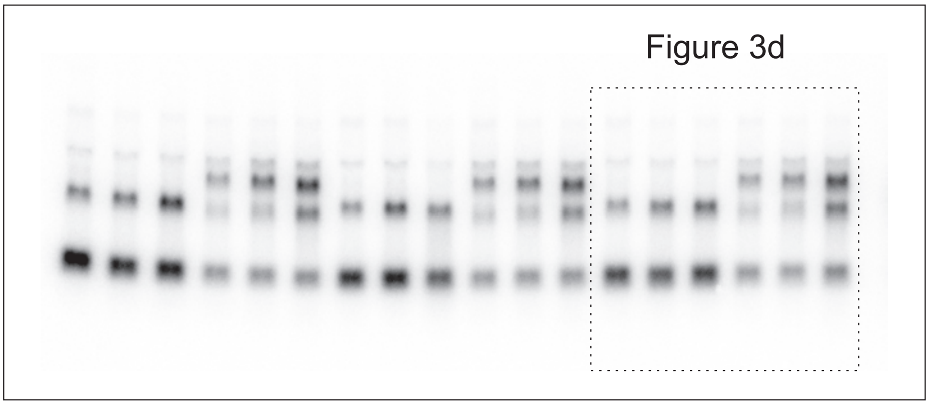

Supplementary Figure 8, continued

Uncropped blots of Figures 1-8 as indicated.

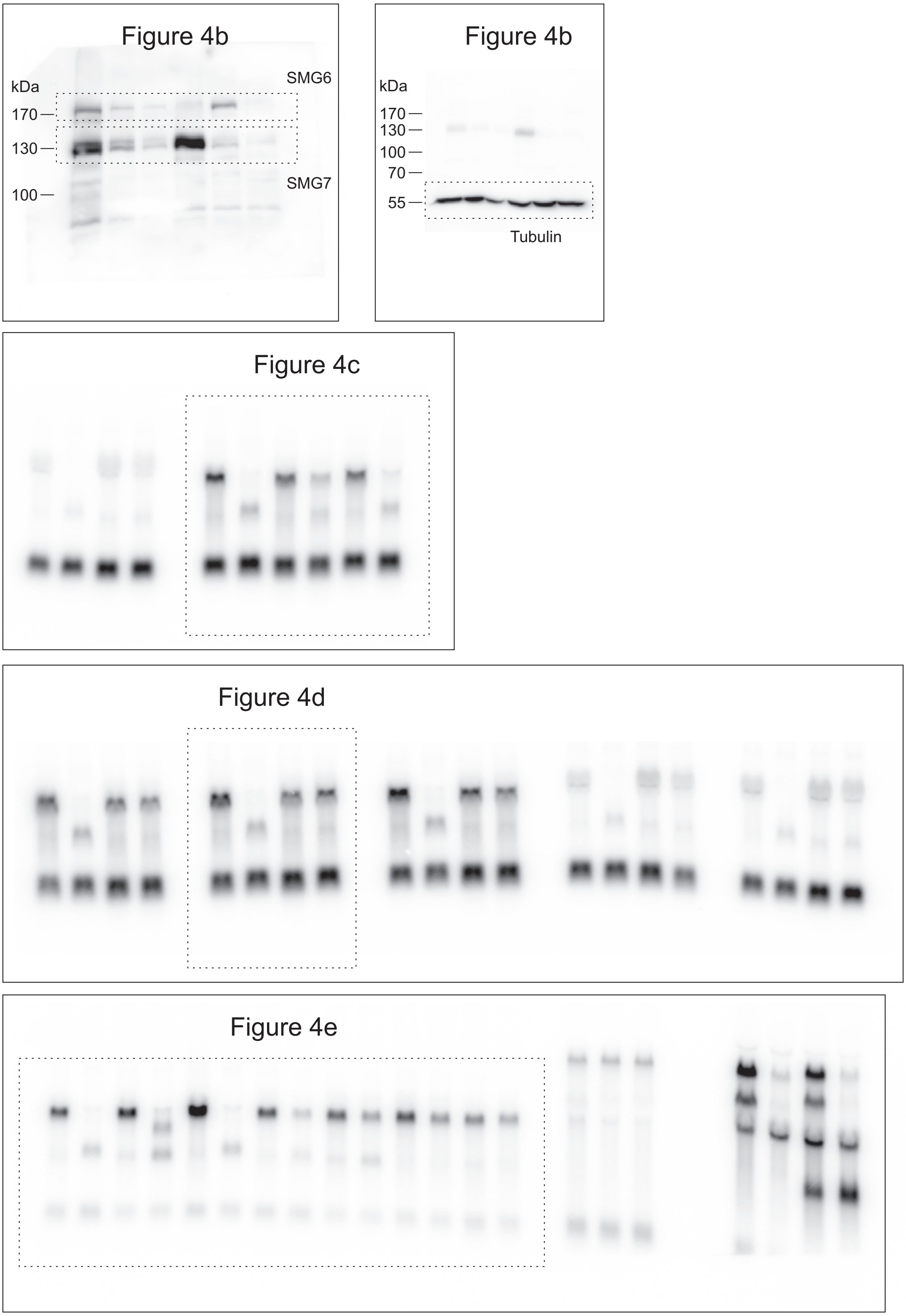

Supplementary Figure 8, continued

Uncropped blots of Figures 1-8 as indicated.

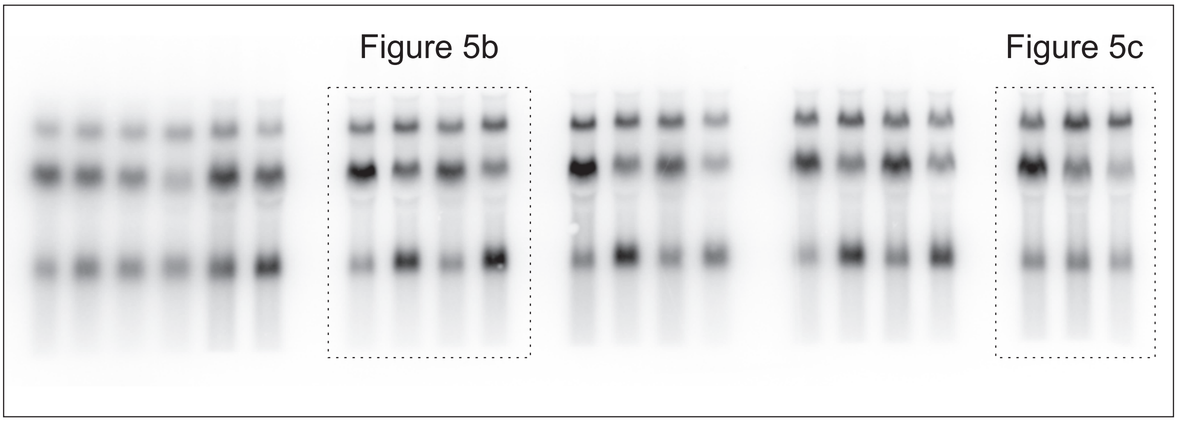

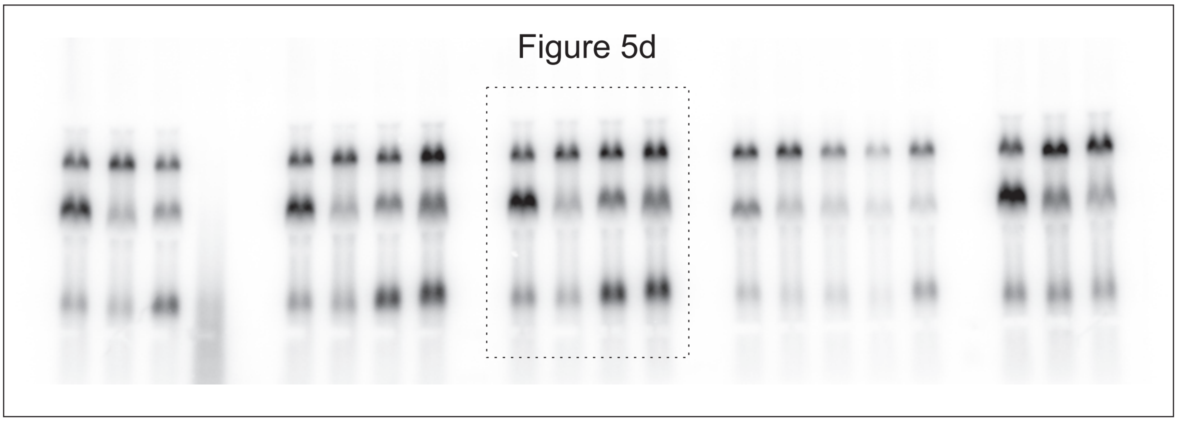

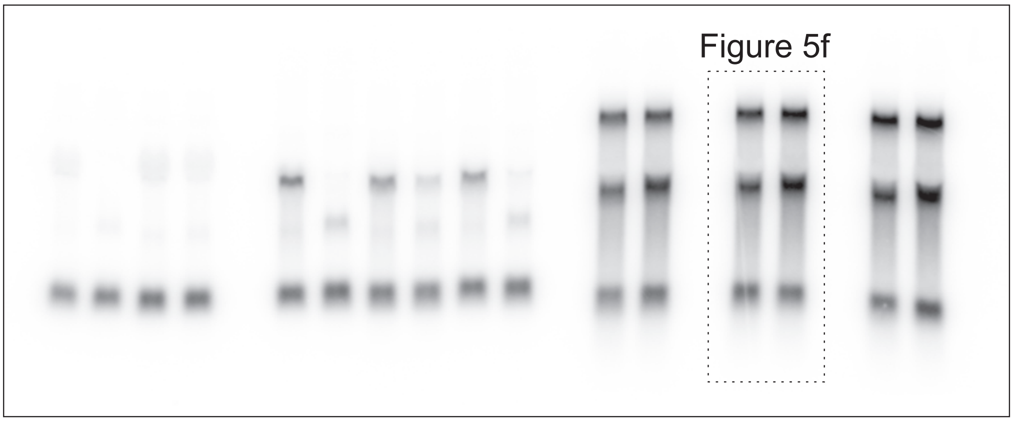

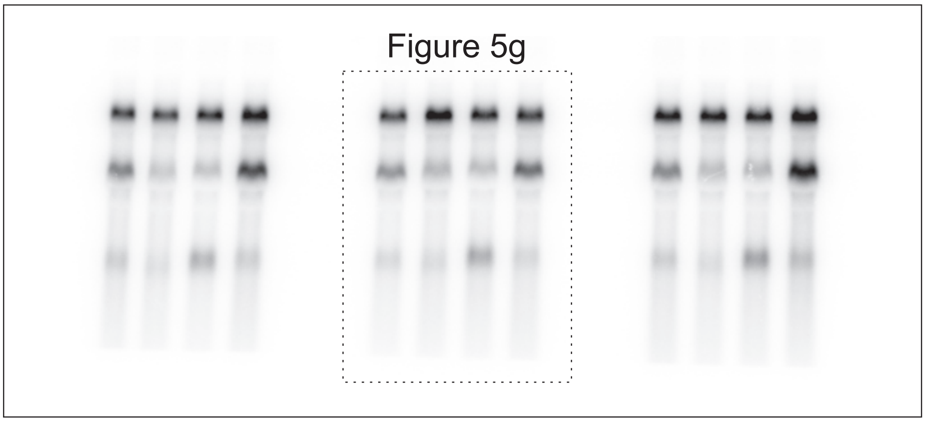

Supplementary Figure 8, continued

Uncropped blots of Figures 1-8 as indicated.

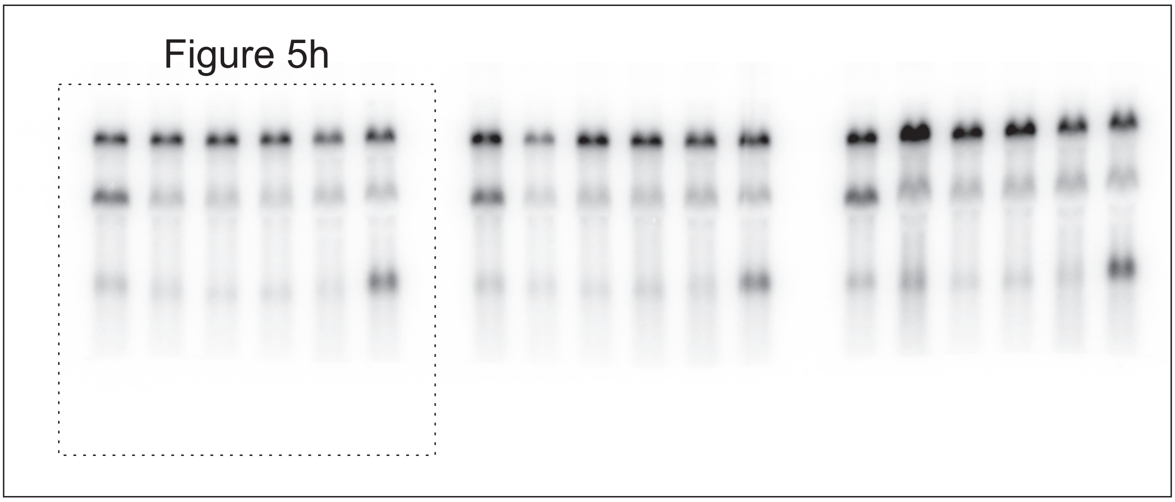

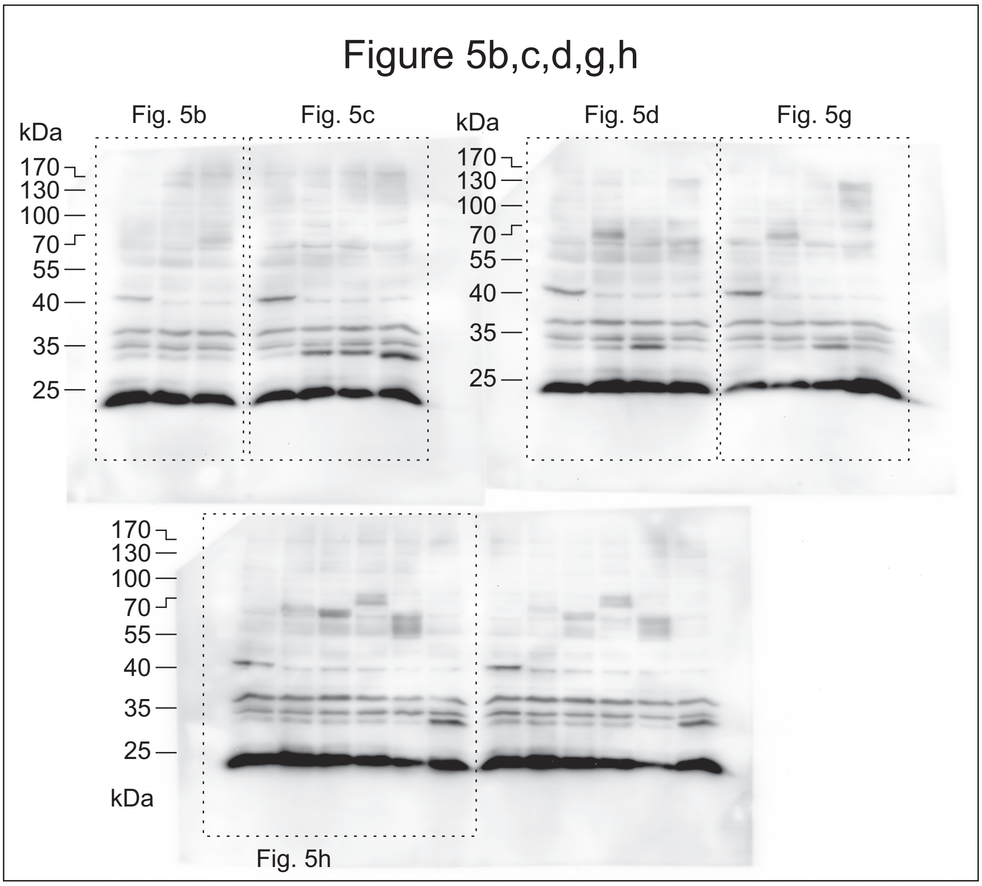

Supplementary Figure 8, continued

Uncropped blots of Figures 1-8 as indicated.

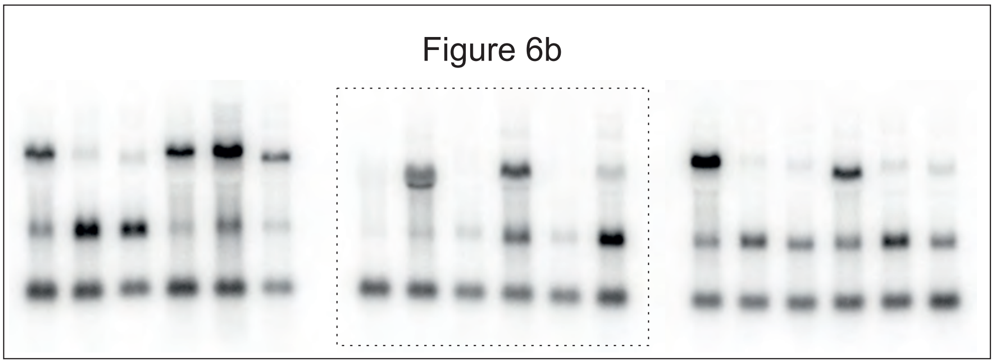

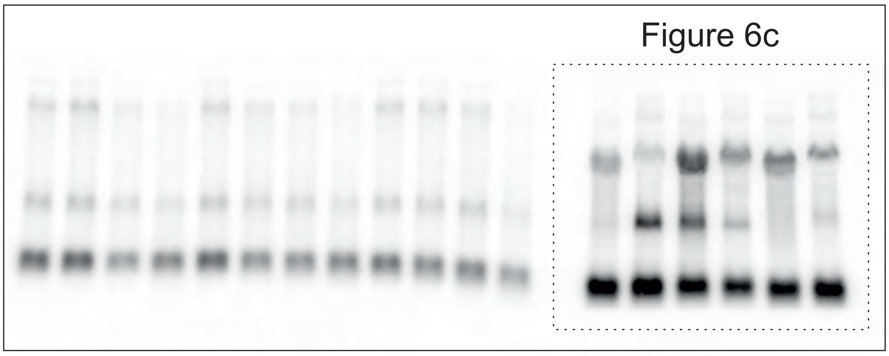

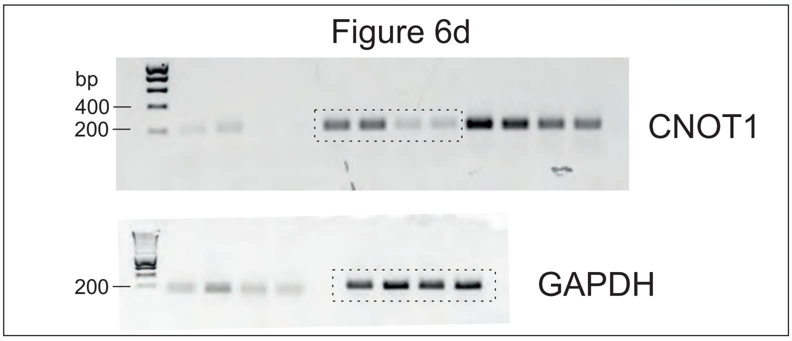

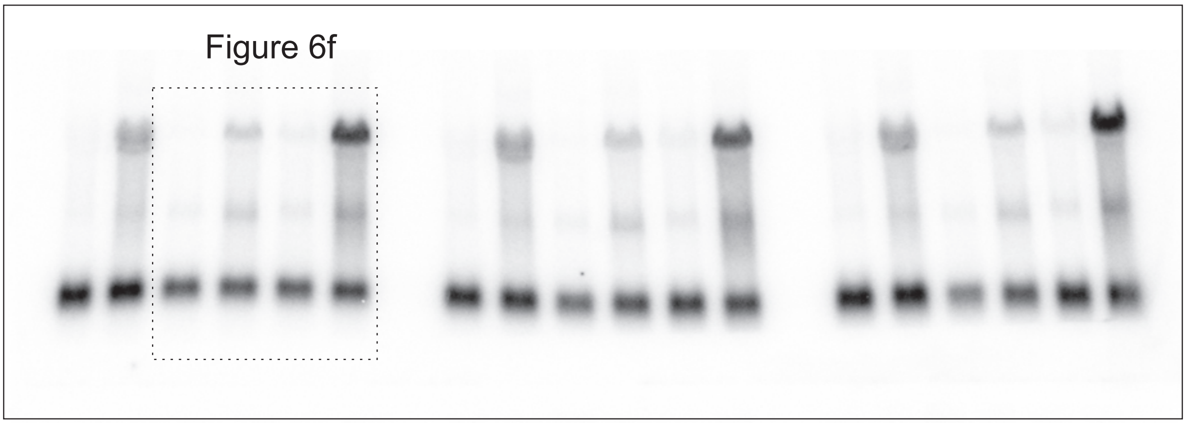

Supplementary Figure 8, continued

Uncropped blots of Figures 1-8 as indicated.

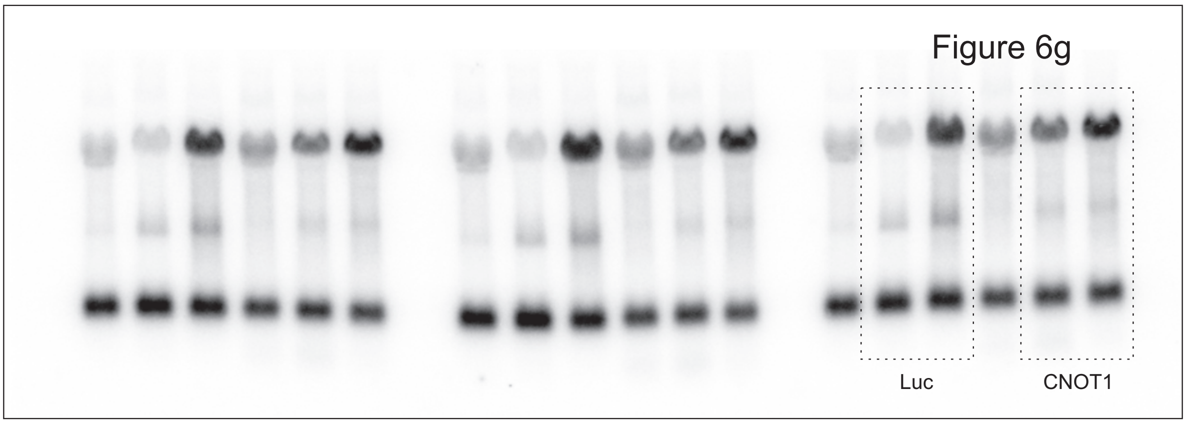

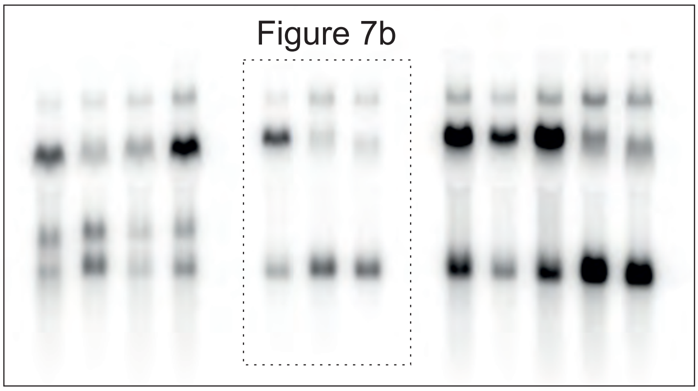

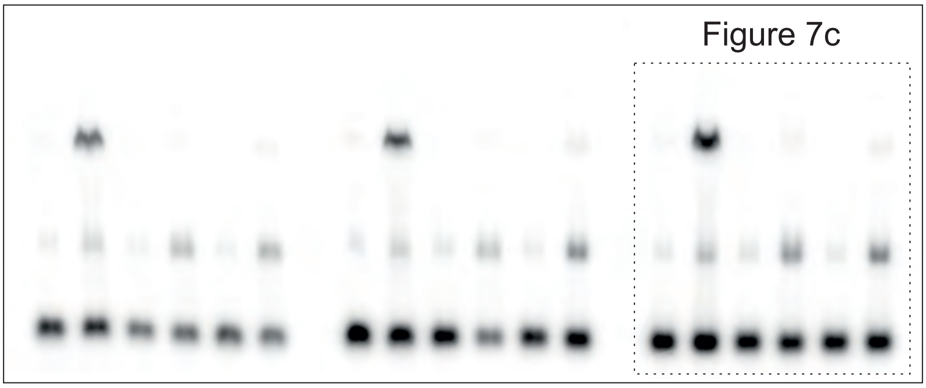

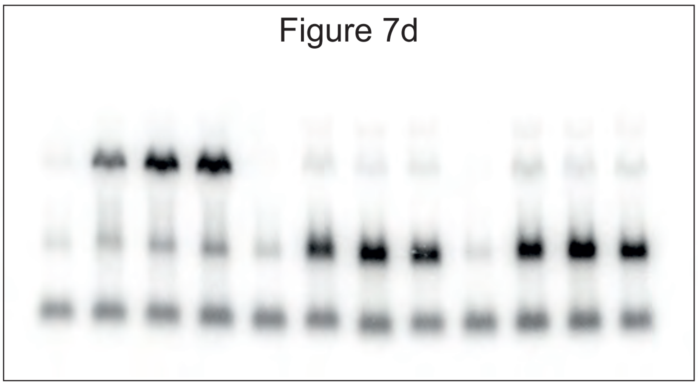

Supplementary Figure 8, continued

Uncropped blots of Figures 1-8 as indicated.

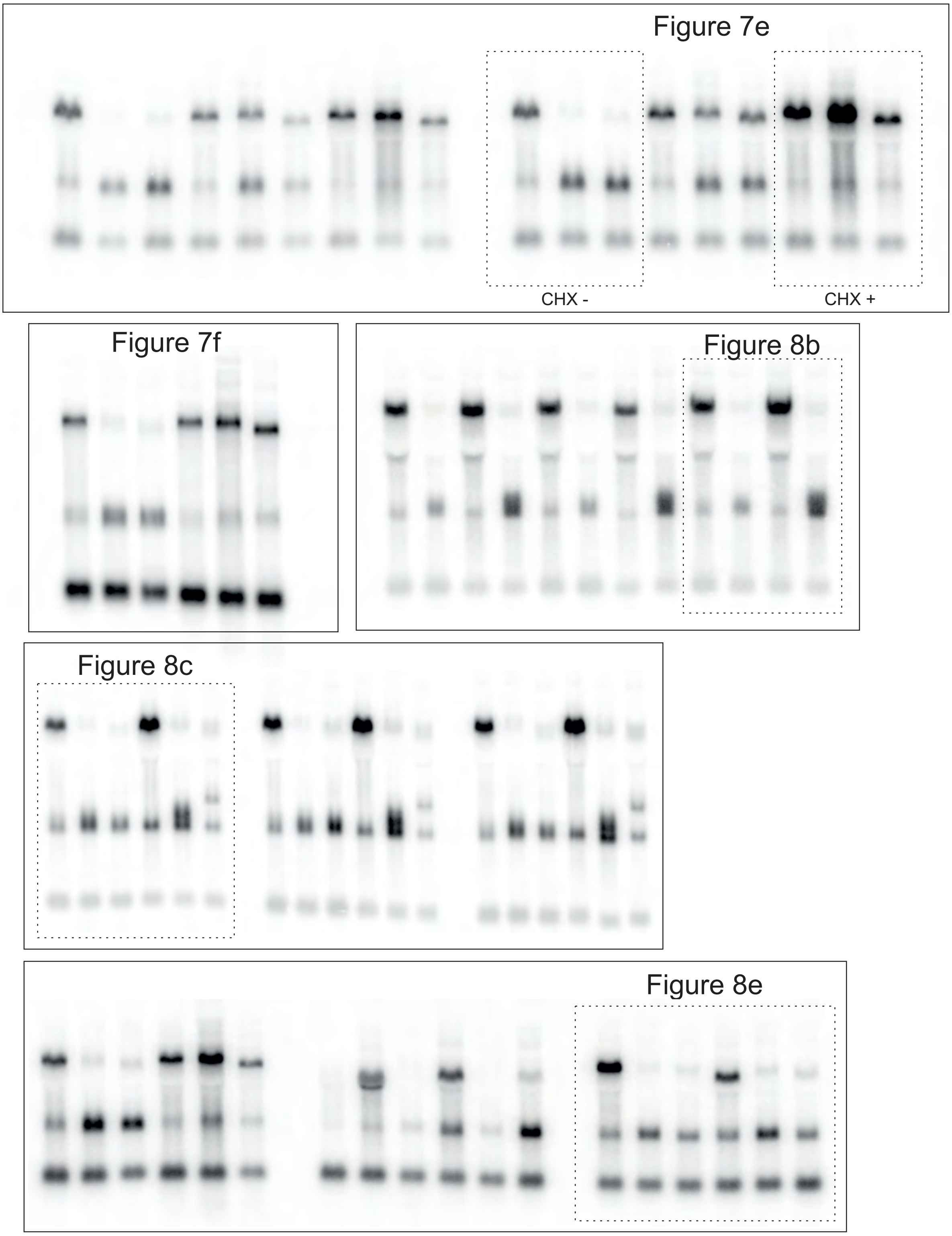

Supplementary Figure 8, continued

Uncropped blots of Figures 1-8 as indicated.

Figure 8f

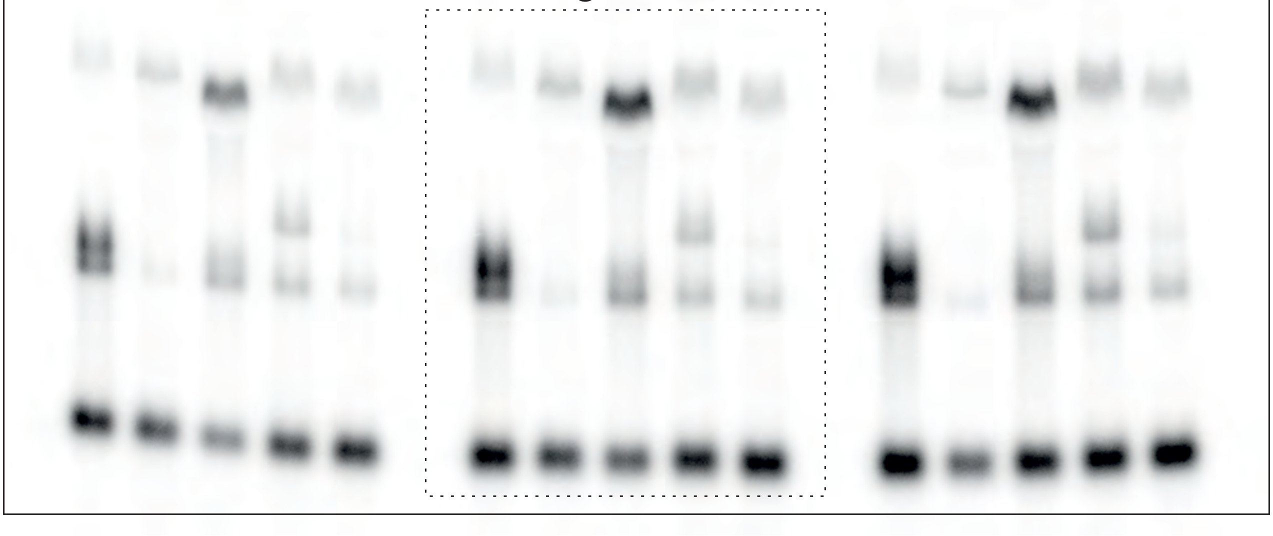

Supplementary Figure 8, continued

Uncropped blots of Figures 1-8 as indicated.

Supplementary References

1. Fallmann, J., Sedlyarov, V., Tanzer, A., Kovarik, P. & Hofacker, I.L. AREsite2: an enhanced database for the comprehensive investigation of AU/GU/U-rich elements. *Nucleic Acids Res* **44**, D90-5 (2016).
